# The role of somatostatin analogues in treatment of TSH secreting pituitary adenomas

**DOI:** 10.1186/1756-6614-6-S2-A64

**Published:** 2013-04-05

**Authors:** Wojciech Zgliczyński, Piotr Zdunowski, Izabella Czajka-Oraniec, Wojciech Jeske, Grzegorz Zieliński

**Affiliations:** 1 Department of Endocrinology, Medical Centre of Postgraduate Education, Warsaw, Poland; 2 Department of Neurosurgery, Military Institute of Medicine, Warsaw, Poland

## Background

TSH-secreting tumours are extremely rare case of hyperthyroidism. Most important clinical feature is preserved TSH level in subjects with apparent thyrotoxicosis. Possible misdiagnosis of primary thyroid hyperfunction could lead to mistreatment with anti-thyroid medications. This worsens disease course and outcome. Neurosurgery success rate is limited by tumour size and its extrasellar expansion. Native somatostatin is key negative regulator of TSH secretion. In most cases tumour cells express somatostatin receptors. This feature creates potential use of somatostatin analogues for medical treatment of TSH-secreting tumours.

## Aim of the study

The aim was to determine potential value of somatostatin analogues in primary TSH-oma treatment. Secondary objective was to evaluate efficacy of long-acting somatostatin analogues in cases after unsuccessful neurosurgery.

## Material

Material comprised of 17 patients with secondary thyrotoxicosis, 7 women and 10 men, aged 20 to 69 years (mean 39), presenting with pituitary macroadenoma (16) and one with microadenoma and empty sella. Before diagnosis was established 8 out of 17 patients received antithyroid medication, in 2 cases strumectomy was performed and 2 patients received 131I therapy.

## Intervention

Somatostatin analogue octreotide long acting repeatable (LAR) administration at least 3 months (3 injections) before surgery, and in cases of unsuccessful surgery stable octreotide LAR therapy.

## Results

Initially, all patients had elevated fT4 and α-SU levels (mean 29.3 pmol/l SD 4.3, and 6.2 ng/ml SD 4.9). 3 months of octreotide LAR therapy led to significant fT4 reduction (to mean 12.2 pmol/l) and TSH reduction from mean 6.5 mIU/ml to 0.3 mIU/l). In all cases clinical improvement was observed.

In 14 out of 17 pre-treated with octreotide LAR decreased tumour volume and in 2 improvement in visual field was observed.

Patients in euthyroid state were referred to neurosurgery department. Transsphenoidal adenomectomy was successful 13 out of 17. In four cases after unsuccessful neurosurgery intervention stable euthyroidism was achieved with octreotide LAR treatment. In one case secondary thyrotoxicosis relapsed 2 years and now is controlled by octreotide treatment.

## Conclusions

Somatostatin analogue treatment is efficient in TSH-secreting tumours in the terms of TSH secretion, thyroid function and clinical improvement. In cases of surgery failure prolonged octreotide treatment could be safe and efficient option of disease management.

